# Two Cytoplasmic Acylation Sites and an Adjacent Hydrophobic Residue, but No Other Conserved Amino Acids in the Cytoplasmic Tail of HA from Influenza A Virus Are Crucial for Virus Replication

**DOI:** 10.3390/v7122950

**Published:** 2015-12-08

**Authors:** Stefanie Siche, Katharina Brett, Lars Möller, Larisa V. Kordyukova, Ramil R. Mintaev, Andrei V. Alexeevski, Michael Veit

**Affiliations:** 1 Institute of Virology, Faculty of Veterinary Medicine, Freie Universität Berlin, 14163 Berlin, Germany; st.siche@googlemail.com (S.S.); katharina.brett@gmail.com (K.B.); 2 Robert Koch Institute, Advanced Light and Electron Microscopy (ZBS4), Nordufer 20, 13353 Berlin, Germany; moellerL@rki.de; 3 A.N. Belozersky Institute of Physico-Chemical Biology, Lomonosov Moscow State University, 119991 Moscow, Russia; kord@belozersky.msu.ru (L.V.K.); ramil.mintaev@gmail.com (R.R.M.); aba@belozersky.msu.ru (A.V.A.); 4 I.I. Mechnikov Research Institute of Vaccines and Sera, Russian Academy of Medical Sciences, 105064 Moscow, Russia; 5 Department of Bioengineering and Bioinformatics, Lomonosov Moscow State University, 119991 Moscow, Russia

**Keywords:** influenza virus, assembly, hemagglutinin, cytoplasmic tail, transmembrane region, acylation, palmitoylation, M1, J0101

## Abstract

Recruitment of the matrix protein M1 to the assembly site of the influenza virus is thought to be mediated by interactions with the cytoplasmic tail of hemagglutinin (HA). Based on a comprehensive sequence comparison of all sequences present in the database, we analyzed the effect of mutating conserved residues in the cytosol-facing part of the transmembrane region and cytoplasmic tail of HA (A/WSN/33 (H1N1) strain) on virus replication and morphology of virions. Removal of the two cytoplasmic acylation sites and substitution of a neighboring isoleucine by glutamine prevented rescue of infectious virions. In contrast, a conservative exchange of the same isoleucine, non-conservative exchanges of glycine and glutamine, deletion of the acylation site at the end of the transmembrane region and shifting it into the tail did not affect virus morphology and had only subtle effects on virus growth and on the incorporation of M1 and Ribo-Nucleoprotein Particles (RNPs). Thus, assuming that essential amino acids are conserved between HA subtypes we suggest that, besides the two cytoplasmic acylation sites (including adjacent hydrophobic residues), no other amino acids in the cytoplasmic tail of HA are indispensable for virus assembly and budding.

## 1. Introduction

Most models of influenza virus assembly postulate a direct interaction between the viral matrix protein M1 and the cytoplasmic domains of the envelope glycoproteins hemagglutinin (HA) and/or neuraminidase (NA) as a driving force for virus assembly [1,2,3]. However, such an interaction has never been demonstrated biochemically, for example by co-precipitation approaches. Experiments to demonstrate that an interaction of HA and NA with M1 by indirect methods yielded contradictory results. M1 is not intrinsically targeted to the assembly site, the apical plasma membrane, but rather localizes to the cytosol, the nucleus and a significant fraction to internal membranes, such as the Golgi [4]. In one report, co-expression of M1 with HA and NA did stimulate the membrane association of the M1 protein significantly [5], but this effect was not seen in other studies [6,7]. Subsequently, detergent extraction at low temperature was used to demonstrate the specific interaction of M1 with HA and NA [8]. Both HA and NA fractionate into detergent resistant membranes (DRMs), whereas M1 expressed alone was soluble, but became resistant when co-expressed with HA and NA. In order to define the minimal number of elements required for virus budding, it was found that HA, when expressed alone, was released as virus-like particles (VLPs) if sialidase activity is provided. Co-expression of NA and M2 increased VLP production. Expression of M1 alone does not produce VLPs, but M1 is incorporated into VLPs if HA is also expressed. Furthermore, when the cytoplasmic tails (CT) were deleted from NA and especially from HA, tailless glycoproteins were included in the VLPs, but M1 failed to be efficiently incorporated [9]. The data are consistent with a model in which the glycoproteins control virus budding by sorting to lipid raft nanodomains and recruiting the internal viral core components.

The most pronounced feature of the cytoplasmic tail of HA is the attachment of fatty acids to (mostly) three conserved cysteine residues [10,11]. Although this modification is usually described as palmitoylation, we found by mass spectrometry of HA from virus particles that two different fatty acids are attached, palmitate (C 16:0) and stearate (C 18:0). Whereas palmitate is exclusively attached to two cytoplasmic cysteine residues, stearate is bound only to a cysteine at the end of the transmembrane domain (TMD) [12]. The hydrophobic modification of HA is essential for virus replication, since (depending on the virus strain) either virus mutants with more than one acylation site deleted show drastically impaired growth or could not be created at all by reverse genetics [13,14,15]. Acylation facilitates raft-association of HA [16] and thus enrichment of the protein in small nanodomains of the plasma membrane [17,18], an observation which might explain why palmitoylation affects both assembly of virus particles and the membrane fusion activity of HA [19]. 

Given the central role of the cytoplasmic tail of HA for virus budding, it is surprising that recombinant virus with tailless HA could be generated by reverse genetics [20]. The resulting virus particles had the same morphology and protein composition as wild-type (wt) virus; only the budding efficiency and infectivity of the virions were slightly reduced. A virus where the cytoplasmic tails were deleted from both HA and NA exhibited more severe defects, especially aberrant morphology and altered genome packaging [21]. The relatively mild phenotype for the virus with tailless HA might be due to the specific constellation of viral genes in these experiments, *i.e.*, HA from the A/Udorn/72 (H3N2) (Udorn) strain in the background of the A/WSN/33 (H1N1) (WSN) strain. It was subsequently shown that deletion of cytoplasmic acylation sites from HA of the Udorn virus is lethal for virus replication, but recombinant virus could be rescued and grew to only moderately depressed titers by expression of M1 from the WSN strain [13]. Thus, a defect in virus assembly caused by a lack of acylation could be compensated by a different M1 protein. Although the molecular basis for this effect is mysterious, it is clear that the cytoplasmic tail is important for virus replication. However, it is not known whether (besides the acylation sites) other amino acids of the tail also affect virus assembly and replication as one would assume if HA mediates specific interactions with M1 to recruit it to the assembly site.

In order to characterize the molecular signal for site-specific attachment of stearate we recently analyzed recombinant WSN viruses containing HA with various mutations in its cytoplasmic tail [22]. Exchange of conserved amino acids in the vicinity of an acylation site had a moderate effect on the stearate content. In contrast, shifting the TMD cysteine to a cytoplasmic location virtually eliminated attachment of stearate, indicating that the location of an acylation site relative to the transmembrane span is the decisive factor for attachment of stearate. More importantly, in this context, is the observation that none of the mutations reduced attachment of fatty acids to HA; each of the cysteine residues were stoichiometrically acylated. Thus, any possible effect of the point mutations on virus replication is not due to reduced acylation of adjacent cysteine residues but an inherent property of the respective amino acids. Therefore, we now analyzed the growth properties, protein and genome composition of these viruses. In addition, we provide a comprehensive sequence comparison of the linker region, transmembrane domain and cytoplasmic tail of all HAs present in the database and report reversions of amino acids we observed during propagation of recombinant viruses.

## 2. Materials and Methods

### 2.1. Amino Acid Conservation Analysis

Influenza A virus HA amino acid sequences were extracted from the Influenza Research Database in March 2014 [23], which contained 33,254 entries of H1 to H17 subtypes with full-length HA sequences. After elimination of redundant sequences, the remaining 17,311 unique sequences were aligned, while 11,543 and 5768 sequences were used for alignment of HAs belonging to group-1 and group-2, respectively. Alignment was done using the MAFFT program, which is based on the FFT-NS2 algorithm [24,25]. To represent the sequence conservation graphically, the WebLogo 3 program was applied constructing amino acid sequence logos [26,27,28].

### 2.2. Cells

Madin-Darby canine kidney (MDCK II, ATCC CRL-2936) and human embryonic kidney (HEK) 293T (ATCC CRL-11268) cells were cultured in Dulbecco’s modified Eagle’s medium (DMEM; PAN Biotech, Aidenbach, German) supplemented with 10% FBS (Perbio, Bonn, Germany) at 37 °C, 5% CO_2_ and 95% humidity, using standard techniques.

### 2.3. Generation of Recombinant Virus

Recombinant influenza A/WSN/33 (H1N1) virus was generated using an eight-plasmid reverse genetics system [29], where each plasmid contains the cDNA of a single viral RNA segment, flanked by suitable promoters. In the HA encoding cDNA segment, the codon for I563 (ATA) was replaced by the isoleucine codon TTG, G557 (GGG) was replaced by the alanine codon GCT and the glutamic acid codon GAA, respectively. Q560 (CAG) was replaced by the glutamine codon GAA. G547 (GGG) was replaced by the serine codon ACG. C554 (TGT) and L559 (TTG) were replaced by the serine codon (AGC) and the cysteine codon (TGC), respectively using QuickChange mutagenesis (Stratagene, Waldbronn, Germany), and confirmed by sequencing (GATC Biotech, Konstanz, Germany). For generation of acylation mutants the cysteine codon TGT (Cys) was changed to AGC (Ser) resulting in Ac1, TGC (Cys) was replaced by TCT (Ser) and TGC (Cys) was changed to AGT (Ser) generating Ac2 and Ac3, respectively.

HEK 293T cells in 60 mm dishes were transfected with the eight plasmids encoding WSN cDNA (1 µg each) using TurboFect (Fermentas/Thermo Fisher Scientific, St. Leon-Rot,) in OptiMEM medium (Invitrogen, Karlsruhe, Germany). At 4–6 h after transfection, the medium was replaced by infection medium (DMEM containing 0.2 % bovine serum albumin (BSA), 0.1% foetal bovine serum (FBS), 2 mM glutamine, 100 U/mL penicillin/streptomycin and 1 µg/mL TPCK-treated trypsin, (Sigma-Aldrich, Taufkirchen, Germany) and incubation was continued at 37 °C. At 48 h after transfection, the supernatants were harvested and cleared of debris by low-speed centrifugation (2000× *g*, 5 min, 4 °C). The supernatant was used to infect MDCK cells for virus propagation. After 1 h of adsorption, the cells were washed with PBS, infection medium was supplied and incubation was continued until a cytopathic effect became evident. The supernatant was then harvested.

### 2.4. Sequencing of HA from Virus Particles

To check for correctness of the HA sequences in the recombinant viruses, RNA was isolated from cleared cell culture supernatants with Invisorb Spin Virus RNA Mini Kit (Stratec, Birkenfeld, Germany) followed by reverse-transcription and polymerase chain reaction (RT-PCR) using HA specific primers and the OneStep RT-PCR kit (Qiagen, Hilden, Germany). Before sequencing, PCR products were treated with the ExoSAP-IT Cleanup Kit (Affymetrix, Halbergmoos, Germany) to remove excess of primers and desoxinucleotide triphosphates (dNTPs), which might hinder the sequencing reaction. Two µL ExoSAP-IT reagent was added to 5 µL PCR product, and incubated at 37 °C for 15 min and heat-inactivated for 15 min at 80 °C. Sequencing was performed by GATC Biotech (Konstanz, Germany).

### 2.5. Plaque Assay

Plaque assays were performed on MDCK cells in 6-well plates according to standard procedures. Briefly, cells were infected with serial tenfold dilutions of the virus supernatants in infection medium, incubated for 1 h at 37 °C, washed with PBS and overlaid with 0.9% SeaKem LE agarose (Lonza, Verviers, Belgium) in Eagle’s minimum essential medium (Lonza, Verviers, Belgium) supplemented with 0.2% BSA, 0.1% FBS, 2 mM glutamine and TPCK-treated trypsin (1µg/mL). After 3 days of incubation, the cells were stained using 0.02% neutral red (Biochrom, Berlin, Germany) in PBS and the plaques were counted. Plaque sizes were calculated from digital images using Image J.

### 2.6. Hemagglutination Assay

HA tests were performed to quantify virus particles by hemagglutination. Freshly drawn chicken blood was washed three times in sterile PBS (centrifugation for 5 min at 2000× *g*), the working solution was adjusted to 0.5%. Two-fold virus dilution series with a final volume of 50 µL were prepared in 96-V-well microtiter plates. Then, 50 µL red blood cell solutions were added per well, mixed and incubated for 30 min at 4 °C. The titer was determined by the last viable lattice structure found.

### 2.7. Electron Microscopy

For negative staining, virus supernatants from infected MDCKII cells were harvested and cleared of debris by low-speed centrifugation (2000× *g*, 5 min, 4 °C). The plaque titer was determined and aliquots containing approximately 10^7^ PfU/mL were mixed with fixative (stock solution of 20% paraformaldehyde (PFA; Roth, No. 0335.3, Karlsruhe, Germany) in 0.5 M HEPES buffer (pH 7.2), which was heated to 60–70 °C for 20 min to shift the equilibrium towards non-polymerized FA) to a final concentration of 2% formaldehyde and incubated for 1 h at room temperature before storing at 4 °C. A drop (10 µL) of the test suspension was placed directly onto a glow-discharged electron microscopy sample support (400 mesh copper grid, covered with a carbon reinforced plastic film). After adsorption for 10 min at room temperature, the grids were washed three times in double distilled water and negatively stained with 1% uranyl acetate. The grids were examined using a JEM-2100 transmission electron microscope (JEOL Corp., Freising, Germany) operated at 200 KV. Micrographs were recorded with a Veleta CCD camera (Olympus Soft Imaging Solutions) at a resolution of 2048 × 2048 pixels.

### 2.8. Growth Kinetics of Recombinant Virus

To assess growth kinetics, MDCK cells were infected with recombinant WSN at a low m.o.i. (0.01 or 0.001). After 1 h of adsorption and washing with PBS, infection medium (DMEM containing 0.2% bovine serum albumin, 0.1% FBS, 2 mM glutamine, 100 U/mL penicillin/streptomycin and 1 µg/mL TPCK-treated trypsin, Sigma-Aldrich) was added. Aliquots were removed from cell culture supernatant after a defined incubation time, cleared of debris and stored at −80 °C before titer determination by plaque assay.

### 2.9. Virus Preparation, SDS-PAGE, Western Blotting and Radioactive Labeling

Virus particles were produced by infecting MDCK cells in a 15 cm dish at an m.o.i. of >2. At 24 h after infection, the supernatants were harvested, cell debris was removed (2000× *g*, 10 min, 4 °C), and the virus was pelleted from the supernatant by ultracentrifugation (Beckman SW-28 rotor, Krefeld, Germany, 28,000 r.p.m., 2 h, 4 °C), resuspended in 100 mL PBS and analyzed by sodium dodecyl sulfate polyacrylamide gel electrophoresis (SDS-PAGE) under non-reducing conditions followed by Western blotting using a polyclonal antiserum against fowl plague virus, which cross-reacts with WSN M1, and horseradish peroxidase (HRP)-coupled secondary antibodies for chemiluminescence detection using ECL Plus substrate (GE Healthcare, Solingen, Germany) and a Fusion SL camera system (Promega, Mannheim, Germany), which detects photons over a wide linear signal-response range. In order to prepare radioactive labelled virus particles the cell culture medium was replaced at 3 h *post infectionem* (p.i.) by DMEM lacking methionine, cysteine and glutamine, supplemented with 0.2% BSA, 0.1% FBS, 5 mM glutamine, 1 µg/mL TPCK-treated trypsin and 0.3 mCi/mL (11.1 kBq) [^35^*S*]-methionine/cysteine (EasyTag™ EXPRE35S35S Protein Labelling Mix; PerkinElmer, Rodgau, Germany). Virus preparations were subjected to SDS-PAGE under non-reducing conditions and fluorography using 1 M salicylate. The dried gel was exposed to X-ray film and bands intensities were analyzed with Bio-1 D software (PeqLab, Erlangen, Germay).

### 2.10. Quantitative Real-Time RT-PCR

In order to analyze an equivalent number of virions the supernatants from infected MDCK cells were adjusted to an HA titer of 2^6^. RNA was then extracted with the Invisorb Spin Virus RNA Mini Kit (Stratec) and cDNA synthesized with the Maxima H Minus First Strand cDNA Synthesis Kit (Thermo Scientific, Braunschweig, Germany). 4 µL 5× RT-Buffer, 1µL dNTPs (10 mM), 1 µL Oligo(dT)_18_ Primer (100 µM), 1 µL Maxima H Minus Enzyme mix and 13 µL RNA were used per sample, which were incubated at 50 °C for 30 min. The reaction was terminated by incubation for 5 min at 85 °C.

The reaction product was directly used in a quantitative RT-PCR with the following primers specific for the influenza A virus M gene segment: Forward primer (5′→3′): AGATGAGTCTTCTAACCGAGGTCG, reverse primer (5′→3′): TGCAAAAACATCTTCAAGTCTCTG (Invitrogen). Real-time RT-PCR was performed utilizing a TaqMan probe, OneTaq DNA polymerase (NEB) and the ICYCLER-IQ5 Multicolor Real Time PCR Detection System (BIO-RAD, München, Germany). One assay with a total volume of 25 µL contained 5 µL 5× OneTaq-buffer, 16 µl RNAase free water, 0.5 µL dNTPs (100 µM), 0.25µL forward and reverse primer (100 µM), 0.75 µL probe (10µM, 6FAM-TCAGGCCCCCTCAAAGCCGA-TMR), 0.25 µL OneTaq DNA polymerase and 2 µL cDNA as template. Temperature profile: 3 min, 95 °C (1×); (10 s, 95 °C; 30 s, 55 °C (annealing and elongation)) (40×). Fluorescence values (FAM) were collected during the annealing step. A standard curve was generated by using serial dilutions of an *in vitro* transcribed, M segment-derived RNA transcript. 

## 3. Results

### 3.1. Sequence Comparison of the Linker Region, Transmembrane Domain and Cytoplasmic Tail between HA Subtypes

**Figure 1:**
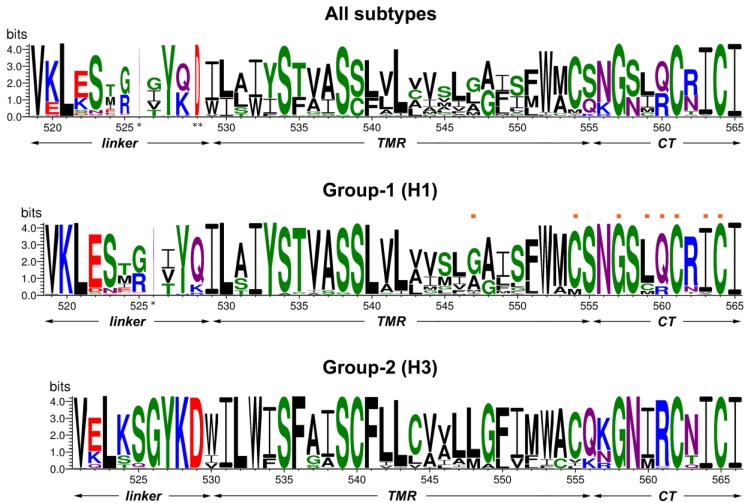
Conservation analysis of the C-terminal region of HA. Every unique HA sequence present in the database was used for the alignment represented by the WebLogo 3.3 server, either all subtypes together (upper panel) or group-1 (middle panel) and group-2 (lower panel) sequences separately. The overall height of the stack indicates the sequence conservation at that position, while the height of symbols within the stack indicates the relative frequency of each amino acid at that position. Gaps found in all HA subtypes (except small samplings of H11, H13, H16 sequences) and in all group-1 subtypes (compared to the group-2 ones) are marked with one or two asterisks, respectively. Amino acid numeration correspond to the HA from A/WSN/33 (H1N1) (UniProt Acc. B4URE7) in the upper and middle panel and to the HA from A/Udorn/72 (H3N2) (UniProt Acc. Q20MD6) in the lower panel. The relative borders of the linker region, transmembrane domain (TMD) and cytoplasmic tail (CT) are indicated. The residues mutated in HA from WSN are marked with orange rectangles above the group-1 logos.

Whereas the crystal structure of the ectodomains of various HA subtypes has been determined, very little is known about the remaining part of the molecule, the cytoplasmic tail, the transmembrane domain (TMD) and the linker region that connects the TMD to the ectodomain. Based on the amino acid sequence as well as on the 3D structures of their ectodomains the 18 antigenic subtypes of HA are divided into two evolutionary groupings: group-1 (H1-group; H1, H2, H5, H6, H8, H9, H11, H12, H13, H16, H17, H18) and group-2 (H3-group; H3, H4, H7, H10, H14, H15) [30,31]. To identify conserved residues, we have extracted every full length HA sequence from the influenza A virus database, deleted the redundant ones and aligned the unique sequences of the linker, TMD and CT region, either together or separated into group-1 and group-2 sequences. The frequency of individual amino acids at each position is listed in Table S1 for above mentioned HA subtypes. To make the wealth of data more comprehensible, they were visualized with the WebLogo 3 program. Results are displayed as logos in Figure 1, either all sequences together or separately for group-1 and group-2 subtypes. Each logo consists of stacks of amino acid symbols, one stack for each position in the sequence. The overall height of each stack indicates the sequence conservation, while the height of each symbol within the stack indicates the relative frequency of the respective amino acid at that position. Thus, the diagram displays the consensus amino acid sequence (top letters at each position of the logo) as well as the amino acid frequencies and the total sequence conservation. Groups of amino acids with similar biochemical properties are displayed by different colors. Negatively or positively charged residues are in red and blue, respectively, large and hydrophilic residues are in purple, neutral amino acids are in green and hydrophobic residues are in black. Note, however, that analysis by alignment of all sequences is biased, since sequences from H1 (~6400), H3 (~4300) and H5 (~2600) subtype are overrepresented in the Flu database, whereas significantly fewer sequences are available for the other subtypes. Therefore, the consensus sequence of each hemagglutinin subtype is displayed in Table S2.

As obvious from Figure 1, the linker region, TMD and cytoplasmic tail differ both by their sequence conservation and types of amino acid residues present. The linker region is the most variable among the three domains, both by its length (10–14 amino acids depending on the HA subtype) and the type of amino acids; positively and negatively charged, polar and hydrophobic residues are abundant. This region is likely to be flexible since it contains the cleavage sites of proteases that were used to enzymatically remove HA’s ectodomain [32,33]. The variable part of the linker region is encompassed by conserved residues. A valine and a leucine located at the beginning and a tyrosine at the end of the linker region are conserved trough all HA subtypes while several other residues are conserved only in group-1 (K520) or group-2 subtypes (526GYKD529). Amino acid numeration through all the text is for the WSN (H1) and Udorn (H3) HA, respectively.

The transmembrane region contains 26–27 amino acid residues and is (by definition) demarcated on both sides by either charged (K, D, E) or large and hydrophilic residues (N or Q). Its N-terminal part that contacts the outer half of the lipid bilayer is highly conserved, both between all subtypes and more pronounced within group-1 and group-2 HAs. The mean conservation value (the relative height of logo stacks) of the N-terminal conserved cluster is ~93% for group-1 (amino acid positions 529–542) and group-2 HAs (531–543). The high conservation of amino acids suggests that this region is not only a membrane anchor (that, in principle, could accommodate many different hydrophobic amino acids) but might play a further, more specific role, for example binding to hydrophobic ligands. In accordance, we have recently identified a cholesterol-consensus motif in group-2 HAs that comprises the completely conserved residues Tyr 527 and Lys 528 in the linker region and Leu 532 and Trp 533 at the beginning of the TMD [34]. In contrast, the C-terminal half of the TMD exhibits much more amino acid variation, a mean conservation value of only 60% for amino acids 543–550 in both group-1 and group-2 HAs was calculated. Up to five different amino acids, sometimes non-conservative substitutions are present at some positions. However, all amino acids are common components of transmembrane regions, and charged residues do not occur.

The cytoplasmic tail (10–11 amino acids) is again highly conserved. Noteworthy, five residues Gly 557, Cys 561, Ile 563, Cys 564 and Ile 565 (WSN numbering) are almost invariant, not only within one group but also through all HA subtypes and are therefore likely to be of functional importance. Ile 563, the palmitoylated C564 and I565 form a hydrophobic patch at the C-terminal end of the tail that is also conserved in HA of Influenza B virus (Table S2). C561 located in close proximity is the second palmitoylated residue in the tail. A completely conserved glycine, an amino acid lacking a side chain, but having a substantial effect on the secondary structure of polypeptides, is present at position 557 in the middle of the cytoplasmic tail. Other residues are not conserved between all HA subtypes but almost invariant within one group. The residue preceding the second acylation site has an amino group in its side chain, which might interact with M1. It is always an arginine in group-2 and either a glutamine or an arginine in group-1 HAs. The beginning of the cytoplasmic tail is defined by hydrophilic residues. In group-1, it is just one residue, a conserved asparagine and in group-2 two hydrophilic amino acids are present, glutamine or lysine followed by lysine, asparagine or arginine. 

The location of the third acylation site is also not completely conserved. However, as is obvious from their consensus sequences, each HA subtype has at least three cysteine residues (Table S2). In most HA subtypes, it is located at the cytosol-facing end of the TMD, at position 554 in group-1 or at position 555 in group-2 HAs. If a cysteine is not present at the end of the TMD, such as in H8 and H9 subtype HAs, a third cysteine is present in the cytoplasmic tail at position 559. Furthermore, H11, H13 and H16 subtypes also have a cysteine at that position but not at position 561. Finally, H11-subtype HAs have even four cysteines, one at the end of the TMD and three in the cytoplasmic tail. We have previously shown that all these cysteines of each HA subtype are stoichiometrically acylated, the ones in the tail exclusively with palmitate, the cysteine at the end of the TMD with (depending on the virus strain and host cell) 30% to 100% stearate [22,35]. Interestingly, if an HA subtype does not have a cysteine at one of the four positions, it is replaced by a hydrophobic amino acid, either by methionine, valine, leucine, isoleucine or phenylalanine suggesting that these positions have the propensity to interact with membranes. Another peculiar feature not immediately obvious from the alignment of sequences is the presence of (at least) one glycine in the variable region of the TMD of almost all (except H4) HA subtypes (Table S2). Glycines are usually not compatible with the α-helical secondary structure, and the transmembrane region likely adopts and thus might change their orientation with respect to the lipid membrane border. 

### 3.2. Mutation of Cytoplasmic Palmitoylation Sites and Non-Conservative Substitution of C-Terminal Isoleucine Prevent Virus Rescue

Based on the described sequence conservation, we created a set of mutants to investigate which amino acids in the cytoplasmic tail of HA are required for or affect virus replication (Table 1). The whole cytoplasmic tail was deleted by replacement of glycine 557 with a stop codon (mutant G557 stop). The same absolutely conserved glycine was also exchanged by the small amino acid alanine (G557A) and by a large, negatively charged glutamic acid residue (G557E). The three cysteine residues at positions 554, 561 and 564 representing the conserved acylation sites were mutated to serine (mutants Ac1, Ac2 and Ac3, respectively). Ac1 was further mutated to introduce a cysteine as putative acylation site instead of leucine at position 559 (mutant Ac1 + L559C). The resulting mutant thus contains an identical location of the three cysteines as in H8 and H9 subtype HAs from naturally occurring avian, equine, swine, and human virus strains. The first of the two isoleucines encompassing the last acylation site at the C-terminus of HA was exchanged by either the similar amino acid leucine (I563L) or by the large, but hydrophilic residue glutamine (I563Q). Furthermore, position 560, which is mainly glutamine in group-1 subtypes (including HA of WSN) and always arginine in group-2 subtypes, was converted to glutamic acids (mutant Q560E). Finally, a glycine present in the TMD of each HA subtype but at a slightly different position (Table S2) was mutated to an isoleucine, a hydrophobic amino acid usually compatible with transmembrane regions of proteins. However, since the codon for isoleucine always reverted to a codon specifying a serine (see below) the mutant is termed G547S. For each mutant, we changed at least two nucleotides at the respective codon to prevent reversion to the wild-type sequence.

**Table 1:** Mutations introduced in the transmembrane region and cytoplasmic tail of hemagglutinin (HA). Amino acid sequence of the transmembrane region and cytoplasmic tail of HA from the A/WSN/33 (H1N1) strain. Acylated cysteine residues are highlighted in grey, introduced amino acid exchanges are in bold. An asterisk indicates a stop codon. + or − indicates whether infectious virus could be rescued by transfection of cells.

Mutants	Transmembrane	Cytoplasmic Tail	Virus
Wild type	LGAISFWMCS	NGSLQCRICI	+
G557Stop	LGAISFWMCS	N *	−
Ac2 (C561S)	LGAISFWMCS	NGSLQ**S**RICI *	−
I563Q	LGAISFWMCS	NGSLQCR**Q**CI *	−
Ac3 (C564S)	LGAISFWMCS	NGSLQCRI**S**I *	−
G547S	L**S**AISFWMCS	NGSLQCRICI *	+
Ac1 (C554S)	LGAISFWM**S**S	NGSLQCRICI *	+
Ac1+L559C	LGAISFWM**S**S	NGS**C**QCRICI *	+
G557A	LGAISFWMCS	N**A**SLQCRICI *	+
G557E	LGAISFWMCS	N**E**SLQCRICI *	+
Q560E	LGAISFWMCS	NGSL**E**CRICI *	+
I563L	LGAISFWMCS	NGSLQCR**L**CI *	+

Having verified the mutation by sequencing, the mutant HA plasmid together with seven plasmids encoding the other viral proteins were transfected into HEK 293T cells and three days later the supernatant was tested for virus particle release by a plaque assay in MDCK cells. For four mutants, HA with deleted cytoplasmic tail (G557stop), HA with deleted C-terminal palmitoylation sites (Ac2, Ac3) and the mutant with a non-conservative substitution in the C-terminal hydrophobic patch (I563Q), we never (five transfections) rescued infectious virus particles, although control experiments using plasmids with wild-type HA done in parallel showed that transfection was successful. Note that the failure to generate infectious virus particles with HA G557 stop and HA I563Q is not due to a complete blockade of acylation since these HA mutants are efficiently labeled with ^3^H-palmitate when they are expressed from a plasmid in CV1 cells [22]. 

In summary, both palmitoylation sites in the cytoplasmic tail, but not the stearoylated cysteine at the end of the TMD of H1 subtype HA are indispensable for virus replication.

### 3.3. Reversion at Three Codons Causing Amino Acid Exchanges during Amplification of Recombinant Virus

**Figure 2:**
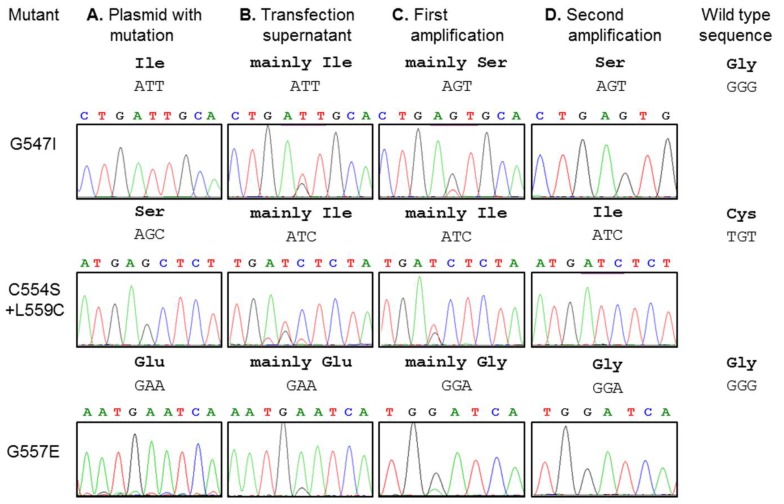
Observed reversions in codons of the mutants G547I, Ac1 + L559C and G557E. (**A**) Sequencing chromatograms of plasmids after site-specific mutagenesis and before transfection into 293T cells; (**B**) Sequencing chromatograms obtained from virus particles three days after transfection of 293T cells; (**C**) Sequencing chromatograms obtained from virus particles propagated at an m.o.i. of 0.1 one day after infection of MDCK cells; (**D**) DNA sequence after a second propagation of the virus in MDCK cells. After 24 h the supernatants were collected, the viral RNA was isolated and subjected to RT-PCR and sequencing. Sequence of the mutated codon, the specified amino acid as well as the respective information for wild type HA is indicated. The reversion of G547I to G547S was always observed (five attempts). Thus, we were not able to obtain stable virus particles with an isoleucine at position 547. Reversion of C554S (Ac1) + L559C to C554I + L559C occurred three times and of G557E to G557G once in five experiments.

The other mutations in HA did not prevent rescue of infectious virus, which was amplified for further characterization. After each amplification step, RNA was extracted from virus particles, cDNA was prepared and ~300 nucleotides at the 5′ end of the HA gene were sequenced to ensure that the mutation is still present. During subsequent passage of recombinant virus in MDCK cells, we observed nucleotide reversions at three of the mutated codons. For the mutant having a substitution in the TMD (G547I), we always (five attempts) observed a single nucleotide exchange from ATT (isoleucine) to AGT (serine). A partial exchange of T to G, reflected by superimposed peaks for the respective bases, was already visible in the viral genomes prepared from the transfection supernatant and the resulting virus G547S completely outgrew G547I after two amplifications (Figure 2, upper row) strongly indicating that it has a competitive fitness advantage. For the mutant with the shifted acylation site (Ac1 + L559C), we observed three times in five experiments that the codon AGC (serine 554) changed to ATC (isoleucine), which outgrew the virus with the desired mutation (Figure 2, middle row). Other amino acid substitutions, which would be possible by a single nucleotide exchange at that codon, *i.e.* to arginine, asparagine, threonine, glycine and surprisingly also to cysteine (specified by another codon (TGC) than the one in wild type HA (TGT)), were never observed. We can only speculate why a reversion to cysteine did not occur but this region in the viral genome is part of a larger signal for packaging of Ribo-Nucleoprotein Particles (RNPs) into budding virus particles [36]. One might conclude that certain nucleotides compromise packaging of the HA encoding segment such that the resulting virus particles are not infectious. The repeated reversion from serine to isoleucine suggests that a long and hydrophobic amino acid at the end of the TMD confers a growth advantage if an acylated cysteine is not present.

Finally, one time in five experiments we observed that in the mutant G557E the codon GAA reverted to GGA. This led to a mutation to glycine, the amino acid present in the wild type HA where it is specified by a different codon. Although substitution of that completely conserved glycine has no drastic effect on virus growth (see below), it apparently confers a fitness advantage since the revertant virus completely outgrew the mutant one in just two amplification steps (Figure 2, lower row).

However, for the last two revertants, we were able to obtain viruses with the desired mutation that were further characterized. The other four recombinant viruses were stable for at least three passages, reversions at the mutated codon or at other sites in the C-terminal ~100 amino acids of HA were never observed. 

### 3.4. Growth Kinetics of Recombinant Viruses

**Figure 3:**
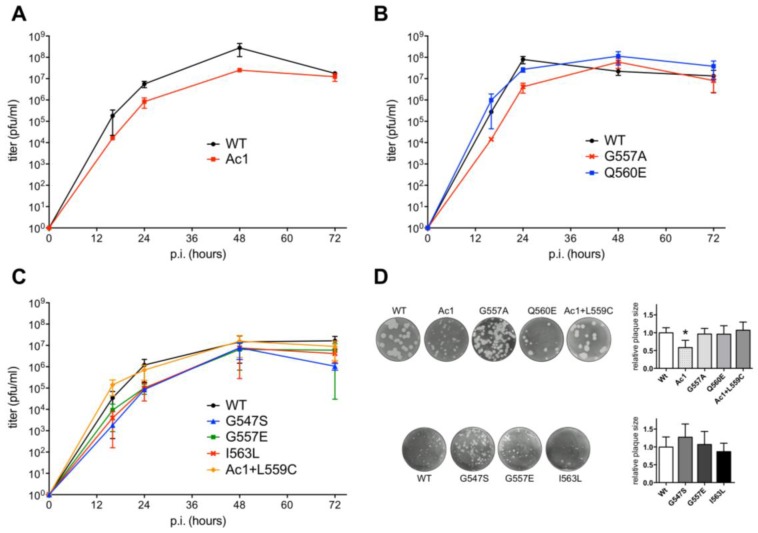
Growth kinetics of recombinant influenza viruses with mutations in HA. (**A**–**C**) MDCK cells were infected with the indicated virus at an m.o.i. of 0.001 (**A**) or m.o.i. of 0.01 (**B,C**). Aliquots were removed from the supernatant at 16, 24, 48 and 72 h p.i. and titers were determined by plaque assay. Experiments were carried out in triplicate and are displayed as means ± standard error. Note that due to the inherent variability of growth experiments titers of wild type (WT) virus somewhat varied between experiments. To account for this variability we always included wild type virus in growth experiments, *i.e.*, all the growth curves shown in one graph were done in parallel. (**D**) Plaque assays of wild type WSN and WSN carrying the indicated HA mutations. Plaque assays of each row were performed in parallel, *i.e.*, with sister cultures infected and stained at the same time. Diameters of 20–40 plaques were measured using ImageJ and normalized to wild-type virus, which was analyzed in parallel. A student’s *t*-test was performed to determine a statistical significant difference between (relative) plaque diameters, However, only the mutant Ac1 produced consistently smaller plaques indicated by the asterisk (*p* < 0.05).

To analyze the replication kinetics of the viruses, MDCK cells were infected with wild type WSN or one of the mutants at a low multiplicity of infection (m.o.i.), and the supernatants were collected at various times and assessed for virus titer by plaque assay. Overall, differences in growth properties between wild type and mutant virus were rather subtle as seen in the growth curves calculated from three independent infections (Figure 3A–C). For the mutant with the deleted acylation site located at the end of TMD, titers were reduced by ~one log at each time point and accordingly Ac1 produced smaller plaques (Figure 3D). This is in perfect agreement with results previously published for the same mutant of H7 subtype HA and also with HA from WSN virus generated with a less efficient reverse genetics system [14,15]. Titers of the mutants with exchanged glycine residues in the TMD (G547S), or in the cytoplasmic tail (G557A and G557E) were reduced at most time points, but the titer difference to wild type did not exceed 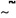 1.5 logs. Calculating the plaque forming unit (PFU) to HA titer ratio revealed that the relative infectivity of the mutants was reduced to 0.8 (G547S), 0.7 (G557A), 0.2 (AC1) and 0.01 (G557E) relative to wild type virus (1.0). The other mutants showed (almost) identical titers to wild type virus, no reduction in specific infectivity (I563L and Q560E = 1.8); and no difference in plaque size was obvious between these mutants and wild type WSN.

**Figure 4:**
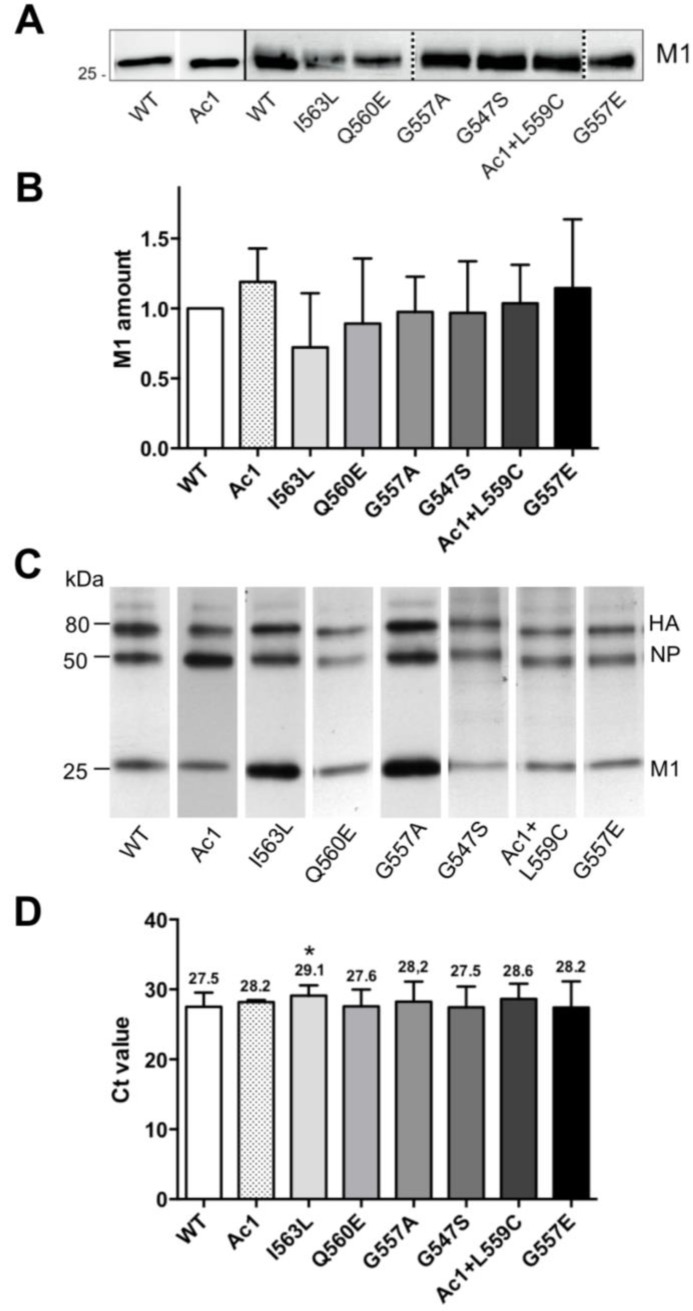
M1 and RNP content of recombinant virus particles with mutations in HA. MDCK cells were infected with an m.o.i. of four. After 24 h, the cell culture supernatant was harvested and adjusted to a HA titer of 2^6^. (**A**) Aliquots were subjected to SDS-PAGE and Western blotting to detect M1. Solid lines between lanes separate different blots, whereas samples separated by dashed line were run on the same gel, but irrelevant lanes were removed; (**B**) M1 bands were quantified by chemiluminescence, normalized to wild type and results from three virus infections are displayed as mean ± SD. A Student’s *t*-test (unpaired, two-tailed) revealed no significant difference in the HA/M1 ratio between wild type and mutant virus particles; (**C**) Protein composition of virus particles metabolically labelled with [^35^*S*]-methionine. Band intensities from this film were quantified by densitometry and revealed the following results for the relative abundance of viral proteins: WT: HA 32%, NP 34%, M1 34%; Ac1: HA 27%, NP 43%, M1 30%; I563L: HA 20%, NP 20%, M1 60%; Q560E: HA 35%, NP 31%, M1 34%; G557A: HA 32%, NP 39%, M1 29%; G547S: HA 40%, NP 40 %, M1 20%; Ac1 + L559C: HA 32%, NP 41%, M1 27%; G557E: HA 32%, NP 39%, M1 29%; (**D**) Real-time PCR with M1 specific primers. The mean C*t* value ± SD is shown for wild type and each mutant and was determined from three independent virus infections. A Student’s *t*-test (unpaired, two-tailed) revealed a significant difference in the HA/RNP ratio between wild type and mutant virus particles only for the mutant I563L, which is indicated by an asterisk (*p* < 0.05).

Since the cytoplasmic tail of HA is supposed to recruit M1 to the budding site, we determined the proportion of both proteins incorporated into virus particles. In order to do so, we adjusted culture supernatants from virus infected MDCK cells to a HA titer of 2^6^ and subjected aliquots to SDS-PAGE and Western blotting with polyclonal antiserum against fowl plague virus, which cross-reacts with WSN M1 (Figure 4A). Quantification of M1 bands by chemiluminescence imaging from three different virus infections revealed that the amount of M1 is increased relative to HA in the mutants Ac1 and G557E and decreased in I563L and Q560E, but the calculated means are not statistically significant different from wild type. The large error bars of the standard deviation suggest that there is a rather large variance between virus preparations (Figure 4B).

In order to estimate the relative amount of proteins incorporated into virus particles we metabolically labelled virus infected cells with [^35^*S*]-methionine, prepared virions from cell culture supernatants and subjected them to SDS-PAGE and fluorography. The resulting X-ray film and quantification of bands showed that the main proteins of virus particles, HA, NP and M1 are present in (roughly) equal proportions in the virus preparation from wild type virus and from the mutants Q560E, G557E and Ac1 + L559C. The mutants G557A and I563L contain more M1 (~60%) at the expense of both HA and NP whereas M1 is reduced (~20%) and both HA and NP are increased in G547S. In contrast, Ac1 contains more NP (43%) and less M1 and HA. Although the effects seem to be large for some mutants we want to express our skepticism about the reliability of the data since even for wild type virus the relative ratios of HA, NP and M1 varied between different preparations (HA: 25%–35%; NP: 35%–45%, M1: 30%–42%, data not shown, see also discussion).

Finally, to quantify the amount of RNPs (and hence of the viral genome) present in virus particles, we performed quantitative real time PCR with M1 specific primers on virus samples adjusted to a HA titer of 2^6^ (Figure 4C). The results from three different virus preparations revealed that two mutants have the same (G547S, Q560E) or a slightly higher cycle threshold (C*t*) value (28.2–28.6) compared to wild type WSN (27.5). However, a statistically significant difference was obtained only for the mutant I563L that has a C*t* value of 29.1. One cycle difference in the C*t* value indicates a two-fold difference in the amount of DNA suggesting that the content of RNPs in mutant I563L is reduced to ~30% compared to wild type. 

## 4. Discussion

In summary, we have shown here that the cytoplasmic tail of H1 subtype HA is essential for virus replication since WSN virus with tailless HA could not be generated. This is in contrast to a previous study, where viruses containing tailless HA from the Udorn strain (H3 subtype HA) could be rescued in the background of a WSN helper virus and even revealed only little growth defects [20]. Except for the two cytoplasmic acylation sites, the C-terminal isoleucine residues and the glycine, the tail sequences of HA from Udorn (KGNIRCNICI, stop codon was introduced instead of the K) and from WSN (NGSLQCRICI, stop codon was introduced instead of the G) are different which might explain the different results. In addition, it might be possible that M1 (or any other protein) that was provided from WSN might allow rescue of otherwise non-infectious particles, as described for HA from Udorn having deletions of acylation sites [13]. 

Individual amino acids in the tail of HA from WSN had very different effects on virus growth. Deletion of the two cytoplasmic palmitoylation sites and non-conservative substitution of the adjacent isoleucine by glutamine completely prevented rescue of infectious virus (Table 1). This is in agreement with earlier studies using a less efficient reverse genetics system where rescue of infectious WSN virus was not possible if Cys 560 or Cys 563 were mutated to serine. However, virus particles could be generated if Cys 560 was exchanged by hydrophobic amino acids, such as alanine and especially with tyrosine and phenylalanine [15]. Also in accordance with our findings are published results using reverse genetics with two other influenza virus strains. Recombinant Udorn (H3 subtype HA) and fowl plague virus (H7 subtype HA) where HA´s two cytoplasmic acylation sites were substituted by serines could be generated, but the resulting virus particles were greatly growth compromised [13,14]. Likewise, although the cytoplasmic tail of HA (including two palmitoylated cysteine residues) from Influenza B virus is not absolutely required for virus replication, the resulting virus revealed defects in replication [37].

**Figure 5:**
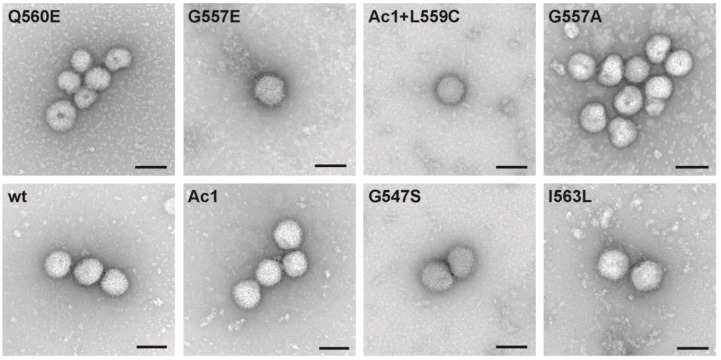
Characterisation of WSN wild type and mutants by negative staining transmission electron microscopy. Representative negative stain images of the indicated virus particles harvested from the supernatant of infected MDCK cells. Scale bars = 200 nm. Differences in particle appearance are not due to morphological changes but to slight modifications of microscope settings and staining conditions of the virus suspensions. Occasionally, filamentous and aberrant formed particles were also observed in each virus preparation, the latter might be an artefact of ultracentrifugation [39].

In contrast, recombinant viruses with mutations in other conserved amino acids showed no altered morphology of virus particles (Figure 5), some only subtle defects in growth kinetics (Figure 3) and M1 and RNP content compared to wild type virus (Figure 4). The standard methods we used, such as HA-assays and metabolic labeling experiments or Western-blotting, are at best semi-quantitative and have an intrinsic error margin which probably exceeds possible small differences between wild type and mutant viruses. Thus, more precise methods, such as quantitative mass spectrometry are required to reliably demonstrate differences in the protein composition of wild type and mutant virus particles [38]. The pleomorphic morphology of Influenza virus particles is a further obstacle that prevents rapid progress in the field. Assuming that every (infectious) particle has eight different RNP segments, the number of NP molecules (and also PA, PB1, PB2) is the same in each virion. However, since the membrane’s surface area varies between particles having a different morphology the number of membrane proteins (and thus the ratio of internal to external viral proteins) most likely varies between individual virions. In addition, at least 90% of particles present in a virus preparation are not infectious, but a shift in the ratio of infectious to non-infectious particles is only insufficiently described by calculating the PFU to HA titer ratio. Thus, new and more precise methods need to be developed to understand the complex interplay between viral proteins that leads to virus assembly and bud formation.

However, although all effects are rather small (compared to the essential nature of the cytoplasmic palmitoylation sites and adjacent hydrophobic residues), the repeated occurrence of amino acid reversion suggests that conserved residues in the tail and in the TMD confer a growth advantage (Figure 2). The most striking example is the repeated and rapid occurrence of an amino acid reversion to serine if a conserved glycine in the TMD was exchanged to isoleucine, a typical constituent of transmembrane domains. Since the other possible single nucleotide exchanges in that codon would specify either a hydrophilic residue (asparagine) or hydrophobic amino acid (isoleucine, leucine, phenylalanine, valine, threonine, methionine), the latter are typical constituents of transmembrane regions, the repeated reversion to serine strongly suggests that a small amino acid is preferred at that position. The transmembrane region of HA is likely to be α-helical [40] and when the inner part of the TMD (541VL**V**VSL**G**AI**S**FWM**C**554) is plotted as a helical wheel, G547 is located together with other small amino acids (V543, S550) on the same side of the helix as the stearoylated cysteine 554. One might speculate that these small residues are located on the outer surface of the trimeric TMD where they might form a groove which binds stearate to align it with the TMD helix as previously speculated [41]. Alternatively, glycine residues are often located at the interface between oligomeric transmembrane regions where they mediate helix-helix interactions [42]. In both cases the substitution of the small glycine residue (amino acid volume of 70 Å^3^) by the large residue isoleucine (volume of 170 Å^3^) might disrupt the assumed interaction and, therefore, a spontaneous reversion to serine (volume of 100 Å^3^) confers a growth advantage.

**Figure 6:**
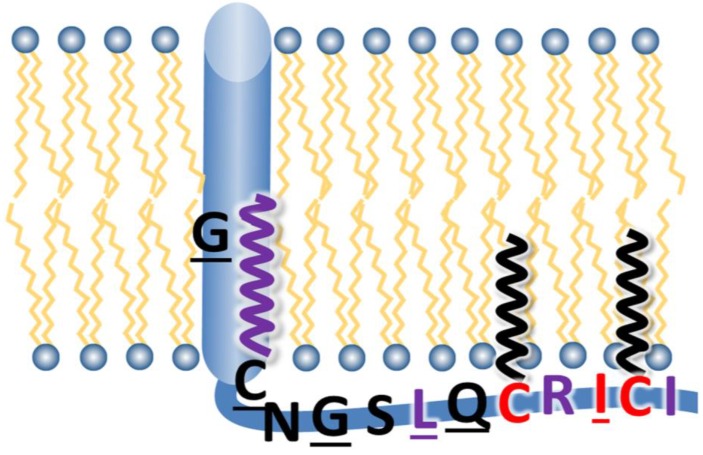
Model of the cytoplasmic tail of HA. Amino acids, indicated in single letter code, which were exchanged in this study, are underlined. Essential amino acids, *i.e.*, the non-conservative substitution of which prevented rescue of infectious virus particles, are in **red**. Fatty acids attached to CT and TMD cysteine residues are depicted by a **black** (palmitate) or **purple** (stearate) zigzag line. Hydrophobic or positively charged amino acids that probably interact with the acyl chains or head groups of (negatively charged) lipids are in purple.

However, our results are difficult to reconcile with the assumption that the cytoplasmic tail of HA alone recruits M1 to the viral assembly site, at least if this is assumed to occur by hydrophilic binding forces, such as salt bridges or hydrogen bonds. The hydrophobic patch ICI (563–565) at the C-terminal end of the tail plus the second palmitoylation site in close proximity (561) likely anchors the tail to the inner side of the plasma membrane (Figure 6). This assumption is based on the observation that the exchange of the acylated cysteines by serine is lethal whereas their substitution by residues with the propensity to insert into lipid bilayers allows virus rescue [15]. Likewise, substitution of isoleucine 563 by the hydrophilic residue glutamine prevents virus rescue, while its exchange by a long and hydrophobic leucine residue has only a minor effect. In addition, substitution of leucine 559 by a cysteine, which is stoichiometrically used as acylation site [22], reduces the virus growth only slightly suggesting that this residue in the cytoplasmic tail also interacts with membranes. In proximity to these acylated or hydrophobic residues is the conserved basic amino acid arginine 562, which has the capacity to interact with the head groups of negatively charged lipids, abundant components at the inner leaflet of the plasma membrane [43]. Thus, if many amino acid side chains (or attached fatty acids) of the cytoplasmic tail of HA are engaged in interactions with the lipid bilayer, only four, N, G, S and Q, remain to specifically bind to M1 (Figure 6). However, exchange of two of them, G 557 and Q 560 had very little influence on virus growth. This suggests that they do not bind with high-affinity to M1 since one would assume that replacement of an amino acid making an essential contact with M1 would result in a more drastically impairment of virus growth. However, in our mutagenesis study, we concentrated on amino acids conserved between all HA subtypes, and it might be that even non conserved residues might have a substantial impact on virus assembly and budding.

Nevertheless, there is clear evidence that HA of both Influenza A and B virus at least functionally interacts with M1 during budding [13,37]. M1, a hydrophobic protein that has the capacity to partially insert into lipid bilayers [44,45,46], might interact with residues located within the inner (and variable) part of the TMD of HA. This assumption might explain why expression of M1 from WSN virus rescues the otherwise lethal deletion of acylation sites from HA of Udorn virus [13]. The two essential cytoplasmic palmitates might also stick to the hydrophobic surface of M1. Alternatively, HA and M1 might not form protein interactions but promote virus budding indirectly, e.g., by changing the fluidity of the plasma membrane to create a stable assembly site. In addition, the cytoplasmic tail of NA, which is completely conserved through all subtypes, might recruit M1 to the viral assembly site. This is consistent with a recent study using Cryo-EM concluding that expression of M1 and M2 together with either of the viral glycoproteins is the minimal requirement to assemble and release virus-like particles [47] and also with the observation that the cytoplasmic tails of both HA and NA control the shape of virus particles [21].

## Supplementary Materials

The following are available online at http://www.mdpi.com/1999-4915/7/12/6458/s1, Table S1: Sequence comparison of the C-terminal region of HA subtypes, Table S2: Consensus sequence of the C-terminal part of HA subtypes.
