# Supplementary material for: Two Cytoplasmic Acylation Sites and an Adjacent Hydrophobic Residue, but No Other Conserved Amino Acids in the Cytoplasmic Tail of HA from Influenza A Virus Are Crucial for Virus Replication
Source: Viruses. 2015 Dec 8;7(12):6458–75. doi: 10.3390/v7122950 (PMC4690873; doi:10.3390/v7122950)
Supplement: Supplementary file 1 [file viruses-07-02950-s001.zip › viruses-104297-Table S2.pdf]

viruses

MDPI

Supplementary Information

Table S2. Consensus sequence of the C-terminal part of HA-subtypes. Acylated cysteine residues are highlighted in yellow, a glycine in the TMD is highlighted on grey.

	Subtype	Transmembrane Domain												Cytoplasmic Tail										
Group-1	H1	V	V	S	L	G	A	I	S	F	W	M	C	S	N	G	S	L	Q	C	R	I	C	I
	H2	A	I	M	I	A	G	I	S	F	W	M	C	S	N	G	S	L	Q	C	R	I	C	I
	H5	A	I	M	V	A	G	L	S	L	W	M	C	S	N	G	S	L	Q	C	R	I	C	I
	H6	V	G	L	I	I	A	M	G	L	W	M	C	S	N	G	S	M	Q	C	R	I	C	I
	H8	A	I	L	I	A	G	G	L	I	L	G	M	Q	N	G	S	C	R	C	M	F	C	I
	H9	A	M	G	F	A	A	F	L	F	W	A	M	S	N	G	S	C	R	C	N	I	C	I
	H11	A	A	I	I	M	G	F	I	F	W	A	C	S	N	G	S	C	R	C	T	I	C	I
	H12	L	L	M	I	I	G	G	F	I	F	G	C	Q	N	G	N	V	R	C	T	F	C	I
	H13	V	G	L	I	L	A	F	I	M	W	A	C	S	S	G	N	C	R	F	N	V	C	I
	H16	V	G	L	I	L	A	F	I	M	W	A	C	S	S	G	N	C	R	F	N	V	C	I
Group-2	H3	C	V	V	L	L	G	F	I	M	W	A	C	Q	K	G	N	I	R	C	N	I	C	I
	H4	V	A	L	L	L	A	F	I	L	W	A	C	Q	N	G	N	I	R	C	Q	I	C	I
	H7	L	A	I	A	M	G	L	V	F	I	C	I	K	N	G	N	M	R	C	T	I	C	I
	H10	L	A	V	I	M	G	L	V	F	F	C	L	K	N	G	N	M	R	C	T	I	C	I
	H14	V	A	L	I	L	G	F	V	L	W	A	C	Q	N	G	N	I	R	C	Q	I	C	I
	H15	L	A	I	A	M	G	L	I	F	M	C	V	K	N	G	N	L	R	C	T	I	C	I
	Flu B	T	L	M	I	A	I	F	I	V	Y	M	V	S	R	D	N	V	S	C	S	I	C	L
